# Supplementary material for: Culture of Cancer Cells at Physiological Oxygen Levels Affects Gene Expression in a Cell-Type Specific Manner
Source: Biomolecules. 2022 Nov 14;12(11):1684. doi: 10.3390/biom12111684 (PMC9688152; doi:10.3390/biom12111684)
Supplement: Supplementary file 1 [file biomolecules-12-01684-s001.zip › Alva 2022 Supplementary Material.pdf]

Supplementary Material for:

Culture of Cancer Cells at Physiological Oxygen Levels Affects Gene Expression in a Cell-Type Specific Manner

Ricardo Alva, Fereshteh Moradi, Ping Liang and Jeffrey A. Stuart *

Table S1. List of differentially expressed genes (DEGs) in LNCaP cells grown in 18% O2 vs 5% O2

(See Excel file)

Table S2. List of differentially expressed genes (DEGs) in Huh-7 cells grown in 18% O2 vs 5% O2

(See Excel file)

Table S3. List of differentially expressed genes (DEGs) in PC-3 cells grown in 18% O2 vs 5% O2

(See Excel file)

Table S4. List of differentially expressed genes (DEGs) in SH-SY5Y cells grown in 18% O2 vs 5% O2

(See Excel file)

Table S5. Functional annotation terms enriched by DEGs upregulated at 5% O2 in LNCaP cells

(See Excel file). Gene Ontology (GO) terms, Kyoto Encyclopedia of Genes and Genomes (KEGG) pathways, and Reactome pathways, sorted by their p-value, enriched by DEGs upregulated at 5% O_2 in LNCaP cells.

Table S6. Functional annotation terms enriched by DEGs upregulated at 18% O2 in LNCaP cells

(See Excel file). Gene Ontology (GO) terms, Kyoto Encyclopedia of Genes and Genomes (KEGG) pathways, and Reactome pathways, sorted by their p-value, enriched by DEGs upregulated at 18% O_2 in LNCaP cells. Data obtained by analysis using the DAVID database.

Table S7. Functional annotation terms enriched by DEGs upregulated at 5% O2 in Huh-7 cells

(See Excel file). Gene Ontology (GO) terms, Kyoto Encyclopedia of Genes and Genomes (KEGG) pathways, and Reactome pathways, sorted by their p-value, enriched by DEGs upregulated at 5% O_2 in Huh-7 cells. Data obtained by analysis using the DAVID database.

Table S8. Functional annotation terms enriched by DEGs upregulated at 18% O2 in Huh-7 cells

(See Excel file). Gene Ontology (GO) terms, Kyoto Encyclopedia of Genes and Genomes (KEGG) pathways, and Reactome pathways, sorted by their p-value, enriched by DEGs upregulated at 18% O_2 in Huh-7 cells. Data obtained by analysis using the DAVID database.

Table S9. Functional annotation terms enriched by DEGs upregulated at 5% O2 in PC-3 cells

(See Excel file). Gene Ontology (GO) terms, Kyoto Encyclopedia of Genes and Genomes (KEGG) pathways, and Reactome pathways, sorted by their p-value, enriched by DEGs upregulated at 5% O_2 in PC-3 cells. Data obtained by analysis using the DAVID database.

Table S10. Functional annotation terms enriched by DEGs upregulated at 18% O2 in PC-3 cells

(See Excel file). Gene Ontology (GO) terms, Kyoto Encyclopedia of Genes and Genomes (KEGG) pathways, and Reactome pathways, sorted by their p-value, enriched by DEGs upregulated at 18% O_2 in PC-3 cells. Data obtained by analysis using the DAVID database.

Table S11. Functional annotation terms enriched by DEGs upregulated at 5% O2 in SH-SY5Y cells

(See Excel file). Gene Ontology (GO) terms, Kyoto Encyclopedia of Genes and Genomes (KEGG) pathways, and Reactome pathways, sorted by their p-value, enriched by DEGs upregulated at 5% O_2 in SH-SY5Y cells. Data obtained by analysis using the DAVID database.

Table S12. Functional annotation terms enriched by DEGs upregulated at 18% O2 in SH-SY5Y cells

(See Excel file). Gene Ontology (GO) terms, Kyoto Encyclopedia of Genes and Genomes (KEGG) pathways, and Reactome pathways, sorted by their p-value, enriched by DEGs upregulated at 18% O_2 in SH-SY5Y cells. Data obtained by analysis using the DAVID database.

Table S13. Differentially expressed genes (DEGs) at 18% O2 vs 5% O2 regulated by Oct4/Nanog/Sox2 †

Gene symbol	log_2FoldChange ‡	P-value	P_{adj} (Benjamini)
**LNCaP**			
ID3	2.019045293	3.18E-06	0.00285462
SPRY4	4.694046957	5.25E-06	0.00418858
JAM2	-4.209861495	0.00024803	0.04947744
IFITM2	2.227010363	0.00041555	0.06652293
BCAT1	-2.87080601	0.0004663	0.06956105
DPP4	2.106726275	0.00049824	0.07002164

ID4	1.778626887	0.00096029	0.09637908
FBXO15	-1.595171509	0.00132032	0.11395218
FUT9	-3.797754559	0.00213372	0.14098766
EIF4A2	-1.222098261	0.00334506	0.18178335
DPYSL4	1.435758599	0.00371992	0.19261111
ISLR2	6.314448042	0.00417417	0.20008036
ENPP3	-1.767796272	0.00468829	0.21376946
DDIT4	-1.136336313	0.00688438	0.25475665
SULF1	-3.478751086	0.00839028	0.27187494
DUSP4	-1.130468257	0.00888501	0.28147611
GADD45G	-1.089612352	0.01181629	0.32216995
TGM3	-1.213323712	0.01260355	0.32216995
AKAP12	-1.175119762	0.01713187	0.37452487
SMAD7	1.035985082	0.01719626	0.37561113
CTH	-1.062728055	0.01783929	0.38134939
MID1	1.400660221	0.01863969	0.38524951
PNMA2	1.000714111	0.01997306	0.38524951
SLC7A7	-2.325666413	0.02095211	0.38524951
AMPD3	5.807622926	0.02267682	0.38524951
FHOD3	1.043581536	0.02270701	0.38550586
CDX2	-5.640369377	0.04096458	0.4702477
ACTN3	2.338178007	0.04103203	0.4702477
ITGA9	-1.619292218	0.04448909	0.48871233
TMC5	-1.92537999	0.04520218	0.48871233
**Huh-7**			
CD38	3.276553774	6.09E-06	0.00193411
ROR1	2.094721495	2.02E-05	0.00451716
H2AFY2	-1.853072816	2.14E-05	0.00469076
SLC39A4	2.701131436	2.59E-05	0.0053286
SORL1	-1.824917152	3.07E-05	0.0061095
MYCN	1.796482892	4.93E-05	0.00841758
TBX15	-1.786319587	0.00017799	0.02019037
SPRY4	1.557259554	0.00025596	0.02500693
F2RL1	-1.506806952	0.0004648	0.03791058
MYC	1.4711455	0.00051022	0.03940291
POLR3G	1.40800477	0.0012498	0.06604644
FGF17	-6.568166877	0.00143701	0.07073657
STOML1	-1.423419677	0.00238706	0.09795339
SEC11C	1.23723905	0.00316147	0.11761671

NRP2	-1.299566678	0.00332341	0.12144791
AMPD3	-1.821429462	0.0034764	0.12144791
CRYL1	-1.251460574	0.00350751	0.12144791
BMF	-1.207243898	0.004023	0.13205145
CDH5	-1.616595306	0.00409161	0.13205145
FHOD3	-1.401882707	0.0041257	0.13205145
CNTFR	-6.329920506	0.00417417	0.13205145
IFITM2	-1.450193042	0.00503336	0.14966206
PNMA2	-1.21612855	0.00514923	0.1511301
FASN	1.14773084	0.00576129	0.16047847
SI	1.322822591	0.00632904	0.17131866
SCHIP1	2.480690696	0.00786477	0.18953814
COBL	-1.11680326	0.00818403	0.19358613
RPL37A	1.088902797	0.00873845	0.19959373
PCYT1B	-1.270827926	0.00918536	0.20575917
APC2	-1.177312546	0.00990657	0.21549585
EGLN3	-1.185078474	0.01211207	0.23785198
CDX2	-1.030495024	0.01319097	0.24435932
KCNH2	3.462789878	0.01341209	0.24478642
CCDC58	1.036574315	0.01389855	0.24879601
SEMA3E	-1.674308537	0.01665464	0.27980754
GADD45G	-2.431313872	0.01786222	0.28922861
SPP1	1.183753976	0.01882144	0.29852395
GBP2	-1.036223445	0.0238791	0.32080313
TNFSF12	-2.050175298	0.0245106	0.32577711
RCOR2	-1.037177952	0.02723331	0.34302761
GPC4	-1.329309265	0.03069574	0.37270881
KRT17	-5.688055616	0.04096459	0.41764497
ANKRD35	-5.470319559	0.04096459	0.41764497
RARG	-2.149592776	0.04520218	0.43921481
**PC-3**			
CD38	-6.712430311	0.00085757	0.18092268
TEX14	-2.66629468	0.00237037	0.27958547
ENPP3	2.845443754	0.01183243	0.45652822
RIMS2	-5.915356606	0.01273085	0.45652822
NPR1	6.138247332	0.01273085	0.45652822
IL17D	1.540341145	0.01296303	0.46245915
PDK1	-1.029815997	0.01412867	0.48359044
BNIP3	-1.017547309	0.01455383	0.4888116

SFRP1	-2.514008738	0.01786222	0.55070317
TEX19	-2.9575577	0.0348666	0.65463081
SLC11A1	5.73002115	0.04096458	0.65463081
**SH-SY5Y**			
CDYL2	6.113623593	0.00724341	0.54893598
MYO1F	1.935697989	0.00813533	0.57874741
GPRC5B	1.180637041	0.00882918	0.60229547
FBXO15	-1.543478938	0.01085078	0.63974638
KLF5	1.331152499	0.01453282	0.66394978
GPC4	1.013405049	0.01851737	0.73233493
POU5F1	-5.913160628	0.02267682	0.73233493
NPHS1	5.756994082	0.02267682	0.73233493
ALPK3	-1.207430098	0.02456322	0.7616433
A2M	1.057029842	0.03400288	0.84574217
GLI1	1.14631948	0.03658208	0.84574217
SLC11A1	1.665889957	0.04543596	0.8682348

† DEGs were identified as Oct4/Nanog/Sox2 targets using the study by Sharov et al. 2008 (reference [n] in the manuscript). ‡ Negative value indicates upregulation at 5% O_2 while positive value indicates upregulation at 18% O_2.

Table S14. Differentially expressed genes (DEGs) upregulated at 5% O_2 induced by HIF-1α and HIF-2α †

Gene symbol	log_2FoldChange	P-value	P_{adj} (Benjamini)	Regulated by †
**LNCaP**				
MAGED4B	-3.2375008	1.59E-09	7.08E-06	HIF-2A
NDUFA4L2	-3.2561557	1.64E-07	0.00026677	BOTH
CXCL8	-2.9197218	3.41E-07	0.00051219	HIF-2A
PLOD2	-7.7876209	1.61E-06	0.00187217	BOTH
ZP1	-4.055585	5.52E-06	0.00427341	HIF-1A
CRABP2	-4.0126735	7.63E-06	0.00536159	BOTH
DDIT3	-1.9780089	8.18E-06	0.00536159	HIF-2A
GSDMB	-2.154615	8.19E-06	0.00536159	HIF-2A
ZMAT1	-2.0081222	1.61E-05	0.00935362	HIF-2A
C1QTNF1	-2.5204612	3.10E-05	0.0151588	HIF-2A
TMEM74B	-3.7765888	5.75E-05	0.02225264	HIF-1A
VEGFA	-1.6803491	8.11E-05	0.02797959	BOTH
ADORA2A	-2.1885528	0.000239	0.04947744	HIF-2A
JAM2	-4.2098615	0.00024803	0.04947744	HIF-1A
PABPC1L	-1.5597169	0.00025256	0.04999201	HIF-1A

FAM227A	-1.6502879	0.00046857	0.06956105	HIF-2A
TNFSF18	-6.8461386	0.00051726	0.07002164	BOTH
IL32	-1.7440857	0.00065572	0.07953652	HIF-1A
LBH	-2.9993402	0.00082998	0.08930434	HIF-2A
SEC31B	-1.5573676	0.00142297	0.11395218	HIF-2A
CHKB	-2.268317	0.0014361	0.11395218	HIF-2A
AMT	-2.3965699	0.00151665	0.11879146	HIF-2A
ZNF596	-1.4112325	0.00152407	0.11900835	HIF-2A
HIC1	-2.5073926	0.00159155	0.11965281	HIF-2A
PLA2G4A	-2.1634785	0.00172833	0.1261888	BOTH
SGK494	-1.5064385	0.00180576	0.12923878	BOTH
ATF3	-1.325444	0.00187885	0.13179837	HIF-2A
ASNS	-1.3473365	0.00223889	0.14546529	HIF-1A
MST1	-1.2992716	0.00278557	0.16276168	BOTH
CARF	-1.2627193	0.00329108	0.18178335	HIF-2A
CREG2	-6.3657188	0.00417417	0.20008036	HIF-1A
CGAS	-6.3657188	0.00417417	0.20008036	HIF-2A
RAB38	-6.3657188	0.00417417	0.20008036	HIF-1A
NPAS1	-1.6849481	0.0042729	0.20355286	HIF-1A
LONRF3	-1.468977	0.00444838	0.208796	HIF-1A
MUC1	-1.7677963	0.00468829	0.21376946	HIF-1A
STC2	-1.1903277	0.00469924	0.21388674	BOTH
C4orf47	-2.6642359	0.00484911	0.2172233	BOTH
GOLGA8H	-1.9114822	0.00512978	0.22127838	BOTH
GOLGA8B	-1.1926649	0.00524526	0.22127838	HIF-1A
SYT17	-2.973949	0.00524731	0.22127838	HIF-1A
MYO15B	-1.1846881	0.00537145	0.22278661	HIF-2A
JMJD7-PLA2G4B	-1.2459965	0.00539755	0.22301124	HIF-2A
CFAP44	-1.3365767	0.00586348	0.23916631	BOTH
NEK11	-1.4265854	0.00604755	0.24381259	BOTH
RSRP1	-1.1862515	0.00610656	0.24478021	HIF-2A
CFAP70	-1.2052133	0.00615513	0.24518739	HIF-2A
EFEMP2	-1.3629907	0.00644802	0.25368853	HIF-1A
DDIT4	-1.1363363	0.00688438	0.25475665	BOTH
HSF4	-1.1679062	0.00691358	0.25475665	HIF-2A
HOXB5	-1.517797	0.00718394	0.25475665	HIF-2A
TRIM9	-6.2156583	0.00724341	0.25475665	BOTH
COL27A1	-1.2036068	0.00764489	0.26343403	BOTH
CDK5RAP3	-1.1411818	0.0082657	0.27187494	HIF-2A

TAS2R19	-3.4787511	0.00839028	0.27187494	HIF-2A
GOLGA8A	-1.1379831	0.00877133	0.27888736	HIF-1A
CFAP43	-1.5256121	0.00878145	0.27888736	BOTH
DUSP4	-1.1304683	0.00888501	0.28147611	HIF-1A
LFNG	-1.1434778	0.00890365	0.28171729	HIF-2A
CCL2	-1.665963	0.00972615	0.29410014	HIF-2A
NME5	-1.1834294	0.00972846	0.29410014	HIF-2A
CLHC1	-1.2395339	0.00986295	0.29697458	HIF-2A
CSAD	-1.0951811	0.0102905	0.30306521	HIF-2A
CCL20	-2.0289948	0.01081171	0.3150521	HIF-2A
DCHS1	-1.2022681	0.01167139	0.32216995	HIF-2A
CCDC17	-1.490143	0.01169695	0.32216995	HIF-2A
TMEM204	-3.3545476	0.01341209	0.3224625	HIF-2A
ITGB8	-1.6256372	0.01417872	0.33553238	HIF-2A
MT3	-2.4180437	0.01448543	0.33876606	BOTH
PPFIA4	-1.1985248	0.01454091	0.33876606	BOTH
ANKRD36	-1.0876217	0.01607993	0.36047852	HIF-2A
AKAP12	-1.1751198	0.01713187	0.37452487	BOTH
ATP6AP1L	-1.076589	0.01867549	0.38524951	HIF-2A
MAMDC4	-1.0400458	0.0196204	0.38524951	HIF-1A
ATP8B3	-1.0684284	0.01971694	0.38524951	HIF-1A
ANXA2R	-1.8108889	0.01998788	0.38524951	HIF-2A
GOLGA8R	-1.8108889	0.01998788	0.38524951	BOTH
MOK	-1.0380073	0.02105426	0.38524951	HIF-2A
TBC1D3E	-1.6245229	0.02144393	0.38524951	HIF-2A
PTH1R	-1.4023481	0.02211347	0.38524951	BOTH
HECW2	-5.8586239	0.02267682	0.38524951	HIF-2A
CHRNE	-5.8586239	0.02267682	0.38524951	BOTH
ISG20	-1.0343563	0.02351034	0.3927464	BOTH
NDP	-1.7498075	0.02610533	0.41739106	HIF-2A
LOX	-1.1605354	0.02653839	0.4180208	BOTH
ZNF439	-1.4063389	0.02655207	0.4180208	BOTH
LNX1	-1.4063389	0.02655207	0.4180208	HIF-2A
KCNMB3	-1.1677023	0.02704513	0.4180208	HIF-2A
OBSL1	-1.260053	0.02884087	0.43965546	BOTH
SGIP1	-3.0685748	0.0348666	0.46685045	HIF-2A
WDR66	-1.0908701	0.03526717	0.46852783	BOTH
CCDC180	-1.1007546	0.03991628	0.4702477	HIF-2A
MYEOV	-5.6403694	0.04096458	0.4702477	HIF-2A

APCDD1L	-5.6403694	0.04096458	0.4702477	HIF-2A
MAMDC2	-5.6403694	0.04096458	0.4702477	HIF-2A
BCKDHA	-5.6403694	0.04096458	0.4702477	HIF-1A
TBC1D3K	-5.6403694	0.04096458	0.4702477	HIF-2A
CLEC3B	-2.4383647	0.04103203	0.4702477	BOTH
BEX1	-1.0020861	0.04199655	0.47936515	BOTH
OTOGL	-1.5126541	0.04281416	0.48329658	HIF-2A
ADM	-1.6192922	0.04448909	0.48871233	BOTH
RAG1	-1.6192922	0.04448909	0.48871233	HIF-2A
UPB1	-1.6938904	0.04511747	0.48871233	HIF-2A
**Huh-7**				
SLC2A3	-5.7441153	1.17E-25	2.73E-21	BOTH
HOXD11	-8.4712232	3.09E-09	5.28E-06	HIF-2A
IGFBP1	-2.6642416	7.76E-09	1.13E-05	HIF-2A
SLC2A14	-2.6352853	8.68E-09	1.19E-05	HIF-2A
YPEL1	-4.2429854	2.89E-08	3.73E-05	BOTH
TXNIP	-2.7269901	3.41E-08	4.17E-05	HIF-2A
NOXA1	-5.0943301	7.22E-08	7.30E-05	HIF-2A
EFEMP2	-2.4541051	1.10E-07	9.51E-05	HIF-1A
SEMA4F	-2.9959769	7.87E-07	0.00045502	HIF-2A
PNCK	-2.1287437	1.17E-06	0.00061116	BOTH
NPPB	-2.0495175	1.57E-06	0.00068241	HIF-1A
SLC22A17	-7.7659763	1.61E-06	0.00068241	HIF-2A
ADGRG1	-2.5875125	2.08E-06	0.00083356	HIF-2A
LYPD1	-2.9900273	4.15E-06	0.00141771	BOTH
C1QTNF1	-7.3846635	1.96E-05	0.00442038	HIF-2A
HOXD10	-4.3499689	2.30E-05	0.00488621	HIF-2A
CKB	-1.7095559	5.01E-05	0.00843369	BOTH
PPP1R3G	-2.1601664	5.07E-05	0.00848114	BOTH
ADAM19	-1.7852406	6.63E-05	0.01041676	HIF-2A
COL6A2	-2.547778	7.11E-05	0.01080785	HIF-2A
ERO1B	-2.0371913	8.61E-05	0.01191797	HIF-2A
KANK3	-3.0454426	9.22E-05	0.01246393	HIF-2A
SERPIND1	-1.621099	0.00012125	0.01515436	HIF-2A
LOXL2	-1.6562245	0.00012688	0.01552682	HIF-2A
TG	-1.8343152	0.00017801	0.02019037	BOTH
CRELD1	-1.5979931	0.00018633	0.0208293	HIF-2A
PAM	-1.5648106	0.00020712	0.0221929	BOTH
FUCA1	-1.7022418	0.00022664	0.02373746	HIF-2A

NDRG1	-1.5296774	0.00026767	0.02559465	BOTH
NR4A3	-6.8648736	0.00031521	0.0280813	HIF-2A
CRABP2	-2.2603406	0.00041129	0.03455625	BOTH
GOLGA8M	-3.056656	0.0004663	0.03791058	BOTH
NEURL1B	-1.5985815	0.00047959	0.03885545	HIF-2A
ADSSL1	-2.7833559	0.00049536	0.03940291	BOTH
MAPK13	-1.5743933	0.00052301	0.03961233	HIF-1A
DDX58	-1.5316071	0.00054928	0.04119984	HIF-2A
SERINC2	-1.4308339	0.00066512	0.04593921	HIF-2A
TINAGL1	-1.4386536	0.00067455	0.04593921	HIF-2A
CLDN3	-1.610645	0.00067767	0.04593921	BOTH
RORA	-1.628413	0.00078477	0.05162624	BOTH
ANGPTL4	-1.7491657	0.00082685	0.05345904	BOTH
EBF2	-3.7939003	0.00088486	0.05358008	BOTH
LIMCH1	-1.4052341	0.0010586	0.06121078	HIF-2A
SPAG4	-1.417475	0.00107711	0.06121078	BOTH
FGF11	-2.3670347	0.00111107	0.06255331	BOTH
TFR2	-1.3650182	0.00113572	0.06332803	HIF-1A
DCHS1	-1.4017739	0.00114276	0.06343444	HIF-2A
SLC6A8	-1.3596027	0.00114309	0.06343444	BOTH
UNC5B	-1.388895	0.0011579	0.06405491	HIF-2A
CD300A	-2.6546058	0.00118483	0.06451923	BOTH
SPINT1	-1.4344847	0.00121897	0.06515752	HIF-1A
OAS1	-1.4276495	0.00129401	0.06757892	HIF-2A
HOXA13	-1.6061745	0.00132032	0.06836431	BOTH
VLDLR	-1.3787072	0.00137049	0.06988314	BOTH
ENO2	-1.3821202	0.00143915	0.07073657	BOTH
HLA-A	-1.3515824	0.00172485	0.07910495	HIF-1A
FOLH1	-1.310705	0.00199912	0.08887854	HIF-2A
SORBS1	-1.2902233	0.00203418	0.09009272	HIF-2A
TNFSF10	-1.4704232	0.00208425	0.09143968	HIF-2A
PPL	-1.472126	0.00212999	0.09238952	BOTH
CD82	-2.7585294	0.00227587	0.09639067	HIF-2A
LGALS3BP	-1.284449	0.00231365	0.0978126	HIF-2A
DNAH12	-6.4539561	0.00243493	0.09795339	BOTH
EFR3B	-1.342034	0.00247704	0.09930364	HIF-2A
NKX6-1	-2.0304955	0.00255104	0.10139616	HIF-2A
EPB41L1	-1.3262656	0.00267114	0.10491424	HIF-1A
FBLN7	-1.3436067	0.00307569	0.11704719	BOTH

ALDH6A1	-1.27716	0.00310356	0.11753121	HIF-2A
HOXB9	-1.7862077	0.00314483	0.11753121	HIF-2A
PTPRM	-1.2320631	0.00331475	0.12144791	HIF-1A
ST3GAL5	-1.3113071	0.00335849	0.12144791	HIF-2A
CCDC80	-1.2452105	0.00344435	0.12144791	HIF-1A
AMPD3	-1.8214295	0.0034764	0.12144791	HIF-2A
ADGRL3	-1.4986931	0.00347955	0.12144791	HIF-2A
APLN	-2.2949233	0.00348955	0.12144791	BOTH
CD44	-2.8681636	0.00351516	0.12144791	HIF-2A
LHX1	-1.6438334	0.00359568	0.12367872	HIF-2A
GPR137B	-1.3956366	0.00387827	0.13120978	BOTH
DDR1	-1.250289	0.00396103	0.13205145	HIF-2A
ZNF395	-1.21223	0.00407039	0.13205145	BOTH
DNASE2	-1.2328393	0.00408932	0.13205145	HIF-2A
DLL4	-1.3231711	0.0047322	0.14322315	HIF-2A
COPZ2	-1.9212543	0.00512978	0.1511301	HIF-2A
CTSO	-1.2667592	0.00516363	0.1511301	HIF-2A
EMX1	-3.3570506	0.00528154	0.1511301	HIF-2A
TLE2	-1.2071453	0.00534439	0.15228884	HIF-2A
TBC1D2	-1.2226122	0.00576293	0.16047847	HIF-2A
P4HA2	-1.1358759	0.00690116	0.18129586	BOTH
BOC	-6.1942093	0.00724341	0.18129586	HIF-1A
TMEM45A	-1.133628	0.00841555	0.1948987	BOTH
PLEKHA2	-1.1048872	0.00858066	0.19734673	HIF-1A
FBXO32	-1.6485777	0.0087349	0.19959373	HIF-2A
PHEX	-1.1179427	0.00921304	0.20598231	HIF-2A
IL32	-1.0845243	0.00926258	0.20686062	HIF-1A
PPP1R13L	-1.1038326	0.00937736	0.20876383	HIF-1A
PLOD2	-1.0791938	0.0093861	0.20876383	BOTH
TCTN2	-1.1230457	0.00943891	0.2094212	HIF-2A
PAG1	-1.0869237	0.00948661	0.20969066	HIF-2A
KDM5B	-1.08733	0.01025535	0.21957409	HIF-2A
MST1R	-1.5455886	0.0111591	0.23003172	HIF-1A
NEK11	-1.3940244	0.01144291	0.23432727	BOTH
HSPB8	-1.0732145	0.0114584	0.23432727	BOTH
CCL28	-2.5299073	0.01183243	0.2357563	BOTH
EGLN3	-1.1850785	0.01211207	0.23785198	BOTH
PTGS1	-6.0443937	0.01273085	0.23785198	BOTH
OSCP1	-1.6353396	0.01370504	0.24760128	HIF-2A

C1RL	-1.0379112	0.0137154	0.24760128	HIF-2A
PCDH1	-1.4020752	0.01390998	0.24879601	HIF-2A
NEXN	-1.2857987	0.0139261	0.24889284	HIF-2A
ZSWIM5	-1.056521	0.01413434	0.25109185	HIF-1A
SYNPO	-1.0291614	0.01490876	0.25870029	HIF-2A
CYP1B1	-1.6111342	0.01493103	0.25870075	BOTH
PLIN2	-1.0079241	0.01507363	0.26058885	HIF-2A
ISM2	-1.8909911	0.01530539	0.26283679	BOTH
FAM13A	-1.002365	0.01658485	0.27924031	BOTH
PTGIS	-1.0824336	0.01674544	0.28092704	BOTH
NYAP1	-1.5936866	0.01714302	0.28390989	HIF-1A
HOXD8	-1.5206477	0.01833224	0.2935684	HIF-2A
DNAH8	-1.7846219	0.01998788	0.30799153	BOTH
BAIAP3	-1.1657029	0.02030153	0.30799153	BOTH
FMO5	-1.0415034	0.02061942	0.30799153	HIF-2A
ORAI3	-1.1213829	0.02064353	0.30799153	HIF-1A
HOXA7	-1.6287053	0.02109634	0.30799153	HIF-2A
C2CD4B	-1.1014089	0.02236823	0.30799153	HIF-2A
HOXB6	-5.8771985	0.02267682	0.30799153	HIF-2A
WNT10B	-2.0501753	0.0245106	0.32577711	HIF-2A
APOLD1	-1.3177079	0.02510763	0.33055679	HIF-2A
RCOR2	-1.037178	0.02723331	0.34302761	HIF-1A
MYO15B	-1.0206751	0.02770112	0.34778967	HIF-2A
IRF7	-1.2968136	0.03029879	0.3701022	HIF-2A
PYGM	-2.2314073	0.03229224	0.38475882	HIF-1A
ICOSLG	-1.0799134	0.03246705	0.38575565	HIF-2A
DNAH5	-1.1654709	0.03471845	0.39298024	HIF-2A
NYNRIN	-2.8772642	0.0348666	0.39298024	HIF-2A
CYP26A1	-1.2667618	0.03615719	0.4036135	BOTH
HOXA6	-5.4703196	0.04096459	0.41764497	HIF-2A
FAM189A2	-5.4703196	0.04096459	0.41764497	HIF-2A
HHIPL1	-5.4703196	0.04096459	0.41764497	HIF-2A
TNFRSF10C	-5.6880556	0.04096459	0.41764497	HIF-2A
HOXD4	-5.6880556	0.04096459	0.41764497	HIF-2A
GALNT15	-2.2112743	0.04103203	0.41764497	HIF-2A
COL1A2	-1.1690212	0.04261201	0.42892397	BOTH
SYTL2	-1.0378699	0.04339667	0.43431933	BOTH
HSPA1L	-1.2663175	0.04368423	0.43523181	HIF-1A
RARG	-2.1495928	0.04520218	0.43921481	HIF-2A

**PC-3**				
ZMAT1	-3.9479322	0.00037659	0.13299872	HIF-2A
IL1RL1	-1.4465176	0.00078008	0.18092268	HIF-2A
HOXD4	-2.5536676	0.00087774	0.18092268	HIF-2A
IL33	-1.3795645	0.00151522	0.24380332	HIF-2A
GSTT2B	-1.6044964	0.00193324	0.27958547	HIF-1A
CCL2	-3.6284165	0.00213372	0.27958547	HIF-2A
IMPG2	-2.0797861	0.00255104	0.29005933	HIF-2A
ZFP82	-1.4854943	0.00288886	0.30676826	HIF-1A
CA9	-1.9822973	0.00289243	0.30676826	HIF-1A
GOLGA8R	-2.077555	0.00360343	0.33867885	BOTH
KCNT2	-2.4947382	0.00390492	0.34767898	HIF-2A
ZSCAN20	-1.6283243	0.00392803	0.34767898	HIF-2A
PPP1R3C	-6.3682541	0.00417417	0.34767898	BOTH
PTH1R	-3.4374921	0.00528154	0.36485643	BOTH
SEC61G	-1.1361072	0.00646624	0.42158161	HIF-1A
ASNS	-1.4010723	0.00682681	0.42209183	HIF-1A
CCL28	-1.2076744	0.00992735	0.45652822	BOTH
MAGEH1	-1.3063902	0.0124778	0.45652822	HIF-2A
PDK1	-1.029816	0.01412867	0.48359044	HIF-1A
SLC44A4	-1.6104954	0.01417872	0.48459206	BOTH
LHX4	-2.0704529	0.01438022	0.48721616	HIF-2A
BNIP3	-1.0175473	0.01455383	0.4888116	HIF-1A
PIP5KL1	-1.4144247	0.01570992	0.51574999	BOTH
PPFIA4	-1.1787933	0.01587345	0.51965482	BOTH
GGT7	-1.0879893	0.01607993	0.52201557	HIF-2A
TRIM9	-1.0456638	0.01751609	0.55070317	BOTH
HOXD3	-2.1342142	0.01800317	0.55070317	HIF-2A
NPIPA5	-1.1592072	0.02277553	0.55496236	HIF-2A
ADTRP	-1.1152954	0.02420403	0.57725744	HIF-2A
GOLGA8N	-1.1203257	0.03473971	0.65463081	BOTH
CCDC17	-1.2812361	0.03762082	0.65463081	HIF-2A
KCNMB3	-1.0376118	0.03833351	0.65463081	HIF-2A
CACNB4	-5.5082653	0.04096458	0.65463081	HIF-1A
SEC14L5	-5.5082653	0.04096458	0.65463081	HIF-1A
RSPO3	-5.5082653	0.04096458	0.65463081	BOTH
TFEC	-1.1429641	0.04165292	0.66227014	HIF-2A
ASPHD2	-1.0276231	0.04263314	0.67280701	BOTH
HTR2B	-1.955481	0.04399131	0.68042034	BOTH

HOXB4	-1.7447691	0.04520218	0.68107147	HIF-2A
SIRPB2	-1.0599406	0.04726796	0.70400564	HIF-2A
**SH-SY5Y**				
IGF2	-1.7365057	0.00014545	0.13413262	HIF-2A
MAGED4	-1.7098668	0.00026083	0.1709033	HIF-2A
GOLGA8R	-2.9123334	0.00166513	0.37351472	BOTH
KCNH3	-1.8013704	0.00319623	0.45824036	HIF-1A
EPHA8	-1.8013704	0.00319623	0.45824036	HIF-2A
JMJD7-PLA2G4B	-1.4130935	0.00336081	0.45824036	HIF-2A
GRB14	-1.8676714	0.00406065	0.47922007	HIF-2A
ADRB2	-2.9850336	0.00524731	0.51016677	HIF-2A
MAMDC4	-1.1351591	0.00799235	0.57874741	HIF-1A
ABCB6	-1.2460159	0.00827861	0.58559385	HIF-1A
LHX4	-1.2187811	0.00928247	0.62130155	HIF-2A
MYO15B	-1.1173686	0.00936471	0.62512602	HIF-2A
ACKR4	-6.10281	0.01273085	0.63974638	HIF-1A
CIART	-1.4905633	0.01324019	0.63974638	HIF-1A
TBC1D3L	-1.1864792	0.01578289	0.70049588	HIF-2A
AS3MT	-5.9131606	0.02267682	0.73233493	BOTH
SGK494	-1.0423999	0.02351034	0.74857275	BOTH
MN1	-1.3735769	0.02655207	0.77554789	HIF-2A
NPIPA5	-2.5416678	0.02704271	0.77554789	HIF-2A
PPP1R3G	-2.0303612	0.03229224	0.84574217	BOTH
GOLGA8H	-1.3565108	0.03277855	0.84574217	BOTH
ISG20	-1.1048203	0.0391281	0.84574217	BOTH
PPP1R3C	-1.0050413	0.04072566	0.84574217	BOTH
SPTLC3	-5.6947528	0.04096458	0.84574217	HIF-1A
ADTRP	-5.6947528	0.04096458	0.84574217	HIF-2A
CFAP43	-1.2309009	0.04257185	0.86774594	BOTH
GOLGA8K	-2.1110179	0.04399131	0.8682348	BOTH
GCHFR	-1.9316305	0.04520218	0.8682348	BOTH
GALNT4	-1.9316305	0.04520218	0.8682348	HIF-2A

† DEGs were identified as HIF-1/2α targets using data from the study by Downes et al. 2018 (reference [n] in the manuscript).

Table S15. Biological Processes (Gene Ontology), KEGG Pathways, and Reactome Pathways enriched by HIF-regulated DEGs upregulated at 5% O2 in LNCaP cells

(See Excel file). Biological Processes (GO terms), and KEGG and Reactome pathways, sorted by their p-value, enriched by DEGs upregulated

at 18% O_2 in LNCaP cells. Data obtained by analysis using the DAVID database.

Table S16. Biological Processes (Gene Ontology), KEGG Pathways, and Reactome Pathways enriched by HIF-regulated DEGs upregulated at 5% O2 in Huh-7 cells

(See Excel file). Biological Processes (GO terms), and KEGG and Reactome pathways, sorted by their p-value, enriched by DEGs upregulated at 18% O_2 in LNCaP cells. Data obtained by analysis using the DAVID database.

Table S17. Biological Processes (Gene Ontology), KEGG Pathways, and Reactome Pathways enriched by HIF-regulated DEGs upregulated at 5% O2 in PC-3 cells

(See Excel file). Biological Processes (GO terms), and KEGG and Reactome pathways, sorted by their p-value, enriched by DEGs upregulated at 18% O_2 in LNCaP cells. Data obtained by analysis using the DAVID database.

Table S18. Biological Processes (Gene Ontology), KEGG Pathways, and Reactome Pathways enriched by HIF-regulated DEGs upregulated at 5% O2 in SH-SY5Y cells

(See Excel file). Biological Processes (GO terms), and KEGG and Reactome pathways, sorted by their p-value, enriched by DEGs upregulated at 18% O_2 in LNCaP cells. Data obtained by analysis using the DAVID database.
